# Exploring Sequence Characteristics Related to High-Level Production of Secreted Proteins in *Aspergillus niger*

**DOI:** 10.1371/journal.pone.0045869

**Published:** 2012-10-01

**Authors:** Bastiaan A. van den Berg, Marcel J. T. Reinders, Marc Hulsman, Liang Wu, Herman J. Pel, Johannes A. Roubos, Dick de Ridder

**Affiliations:** 1 Delft Bioinformatics Lab, Department of Intelligent Systems, Faculty Electrical Engineering, Mathematics and Computer Science, Delft University of Technology, Delft, The Netherlands; 2 Netherlands Bioinformatics Centre, Nijmegen, The Netherlands; 3 DSM Biotechnology Center, Delft, The Netherlands; 4 Kluyver Centre for Genomics of Industrial Fermentation, Delft, The Netherlands; Universidade de Sao Paulo, Brazil

## Abstract

Protein sequence features are explored in relation to the production of over-expressed extracellular proteins by fungi. Knowledge on features influencing protein production and secretion could be employed to improve enzyme production levels in industrial bioprocesses via protein engineering. A large set, over 600 homologous and nearly 2,000 heterologous fungal genes, were overexpressed in *Aspergillus niger* using a standardized expression cassette and scored for high versus no production. Subsequently, sequence-based machine learning techniques were applied for identifying relevant DNA and protein sequence features. The amino-acid composition of the protein sequence was found to be most predictive and interpretation revealed that, for both homologous and heterologous gene expression, the same features are important: tyrosine and asparagine composition was found to have a positive correlation with high-level production, whereas for unsuccessful production, contributions were found for methionine and lysine composition. The predictor is available online at http://bioinformatics.tudelft.nl/hipsec. Subsequent work aims at validating these findings by protein engineering as a method for increasing expression levels per gene copy.

## Introduction

In industrial enzyme production, high-level protein production and secretion are key requirements. The commercial market value was estimated to be nearly US$ 5 billion in 2009; roughly half of production is accounted for by filamentous fungi [1]. Interest in industrial enzymes is still growing, driven by the increased demand for sustainable production processes and the need to move from a fossil fuel-based to a bio-based economy. This calls for the exploration of novel enzymes, as well as predictable methods for high-yield production processes. The filamentous fungi *Aspergillus niger*, *Aspergillus oryzae* and *Hypocrea jecorina* are the major fungal workhorses in industrial enzyme production, due to their efficiency in producing polysaccharide-degrading enzymes (particularly amylases, pectinases, lipases and xylanases) in high amounts. The genome sequence of the enzyme producing *A. niger* strain CBS513.88 was published in 2007 [2] and compared with a related citric-acid producing strain ATCC1015 in 2011 [3].

Although rational genetic engineering strategies have been developed [4]–[6], including codon optimization, strong promoters etc., protein overexpression is still often an art. Heterologous expression in particular is less successful, often hampered by low production levels [7]. Although protein overexpression, including the secretion process and quality control mechanisms such as UPR-ERAD, has been studied widely [8]–[12], no generic solution to improve heterologous overexpression is yet available. More successful is the use of fusion proteins, at the cost of reduced overall yield due to the production of the fusion partner. We propose another strategy: to re-engineer proteins to better match the cell’s production and secretion machinery. In this paper, we take a first step in this direction.

Our aim is to identify protein characteristics that correlate with the production level of secreted proteins in a library of *A. niger* strains. Ideally, data on protein structure, folding and even post-translational modification and processing, both intracellular and extracellular, should be exploited to enhance our understanding of the cellular processing of successful and unsuccessful candidates. Such data is however limited and expensive to obtain, unattainable for large sets of non-commercial proteins. On the other hand, some of this information is also captured in the protein sequence as such, which therefore should be informative. Using a large and diverse library of protein sequences should allow focus on generic aspects, ignoring protein-specific aspects.

We constructed a unique library of over 2,600 strains to overexpress a selected protein sequence. After transformation using overexpression cassettes, productivity of each strain was screened by shake-flask growth and analysis of the protein composition of the supernatant on gel. Protein production was scored positive when, compared to the mother strain, an additional band on SDS-PAGE gel was observed in the expected molecular weight range; otherwise it was scored negative. Characteristics found to distinguish between proteins in the positive and negative classes may point to sequence features that could be adapted in optimization schemes to further “streamline” proteins that already show good expression, in analogy to what has been achieved with codon optimization, where gene sequences are adapted to match the translational machinery [13].

Statistically significant associations between sequence features and positive and negative class membership can be obtained relatively easy. However, such analyses are typically univariate, considering only individual features. In contrast, machine learning algorithms can combine large numbers of features and by that achieve more optimal prediction performance. Recently, different machine learning techniques have been applied on sequence data to predict protein localization [14]–[16] or protein solubility [17]. A disadvantage of machine learning approaches is that they often result in “black boxes”, not easily providing insight into the properties that are defining for the prediction. With few exceptions [18], [19], sequence-based predictors are rarely interpreted.

We developed a sequence-based predictor for extracellular protein production by *A. niger*, with the explicit goal of interpreting which combinations of features are most predictive. We consider a large number of potentially interesting features and develop predictors for both homologous and heterologous gene expression. Sequence data was found to be predictive for both, although less accurate prediction results were obtained for the heterologous data set. Interestingly, interpretation of the underlying model parameters show that for both data sets similar properties are predictive for extracellular protein production. The trained classifier algorithms are made available in a freely accessible online tool (http://bioinformatics.tudelft.nl/hipsec).

## Methods

### Experimental Setup

Proteins were experimentally tested for high-level production in *A. niger*. Binary success scores were obtained by SDS-PAGE of (at least) triplicate shake-flask samples with strains over-expressing the introduced gene as described below. A positive success score was given when a clear visible band was present, negative otherwise.

#### Strain

The strain used in this work is a recombinant strain derived from DS03043, a progenitor of CBS 513.88, in which the 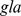
A loci (i.e., the promoter and coding sequences) were deleted, creating the so-called 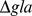
A loci. From this strain, a strain was derived with a strongly reduced production of abundantly secreted proteins by inactivation of the major protease 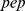
A and a number of alpha-amylases [20]. This protease- and amylase-reduced strain was used as host strain for over-expression of proteins.

#### Molecular biology techniques

In order to obtain targeted integration and expression of any desired gene in the above-mentioned host strain, a standard expression unit was used, where the gene of interest was inserted between the host-own glucoamylase promoter (original 2 kb 5′ 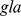
A sequence) and glucoamylase terminator elements (original 2 kb 3′ 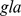
A sequence) in a proprietary *Escherichia coli* vector. The expression unit, a linear piece of DNA, was targeted via single-crossover to the 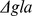
A locus using the homology in the 2 kb 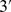
- and direct downstream 2 kb 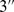-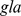
A regions with the identical 2kb-left and 2kb-right flanks of the expression cassette, as described in [20]. All gene sequences were cloned in the *E. coli* vector exactly from start ATG until stop codon.

#### Shake flask fermentations

*A. niger* strain spores were pre-cultured in 20 ml CSL pre-culture medium (100 ml flask, baffle). After growth for 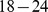 hours at 34°C and 170 rpm, 10 ml of this culture was transferred to Fermentation Medium (FM). Fermentation in FM was performed in 500 ml flasks with baffle with 100 ml fermentation broth at 34°C and 170 rpm for the number of days indicated. The CSL medium consisted of (in amount per liter): 100 g Corn Steep Solids (Roquette), 1 g NaH_2_PO_4

_H_2_O, 0.5 g MgSO_4

_7H_2_O, 10 g glucose

H_2_O and 0.25 g Basildon (antifoam). The ingredients were dissolved in demi-water and the pH was adjusted to pH 5.8 with NaOH or H_2_SO_4_; 100 ml flasks with baffle and foam ball were filled with 20 ml fermentation medium and sterilized for 20 min. at 120°C. The fermentation medium (FM) consisted of (in amount per liter): 150 g maltose

H
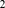
O, 60 g Soytone (peptone), 1 g NaH
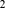
PO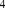 
H
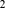
O, 15 g MgSO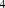 
7H
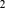
O, 0.08 g Tween 80, 0.02 g Basildon (antifoam), 20 g MES, 1 g L-arginine. The ingredients were dissolved in demi-water and the pH was adjusted to pH 6.2 with NaOH or H
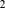
SO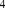; 500 ml flasks with baffle and foam ball were filled with 100 ml fermentation medium and sterilized for 20 min. at 120°C.

#### SDS-PAGE electrophoresis

Sample pre-treatment: 30 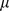
l sample was added to 35 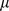
l water and 25 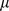
l NuPAGETM LDS sample buffer (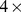, Invitrogen) and 10 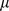
l NuPAGETM Sample Reducing agent (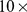, Invitrogen). Samples were heated for ten minutes at 70°C in a thermo mixer. SDS-PAGE was performed in duplicate according to the supplier’s instructions (Invitrogen: 4–12% Bis-Tris gel, MES SDS running buffer, 35 min. runtime). One of the two gels was used for blotting, 10 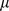
l of the sample solutions and 1 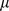
l marker M12 (Invitrogen) were applied on the gels (NuPAGETM BisTris, Invitrogen). The gels were run at 200 V, using the XCELL Surelock, with 600 ml 20 times diluted MES-SDS buffer in the outer buffer chamber and 200 ml 20 times diluted MES-SDS buffer, containing 0.5 ml of antioxidant (NuPAGETM Invitrogen) in the inner buffer chamber. After running, the gels were fixed for one hour with 50% methanol/7% acetic acid (50 ml), rinsed twice with demineralised water and stained with Sypro Ruby (50 ml, Invitrogen) overnight. Images were made using the Typhoon 9200 (610 BP 30, Green (532 nm), PMT 600 V, 100 micron) after washing the gel for ten minutes with demineralised water. Typical detection limit for the fermentation samples using the described method is around 50 mg/l.

### Data

Two protein data sets were tested for high-level production, one for homologous gene expression (Supplementary Table S1) and one for heterologous gene expression. Proteins in the heterologous data set originated from 14 different fungal donor organisms (Supplementary Table S2–S3). All proteins have a signal peptide (length 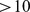 amino acids) as predicted by SignalP 3.0 [21], and a total sequence length longer than 100 amino acids. Proteins containing the most common ER retention signal (C-terminal [HK]DEL) and proteins predicted to be transmembrane by both TMHMM [22] and Phobius [23] were filtered out of the data set.

To avoid biasing subsequent analyses, sequence redundancy was reduced using BLASTCLUST [24]. Two sequences were considered redundant when the aligned sequences shared 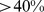 identity over a length of minimal 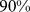 for at least one of the sequences. From the obtained protein clusters, we selected a representative protein, with the shortest average distance to all other proteins in the cluster, and removed the remainder. If a cluster contained proteins with both positive and negative labels, one positive and one negative protein was selected. This resulted in data sets 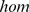 and 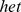 containing 345 proteins (178 positives, 167 negatives) and 991 proteins (163 positives, 828 negatives), respectively.

To train a classifier on 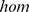 en test it on 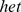, a data set 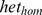 was constructed that contains the 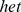 data set without proteins that share 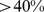 identity with any protein in 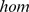. This data set contained 906 (128 positives, 778 negatives) proteins.

### Protein Representations

**Figure 1:**
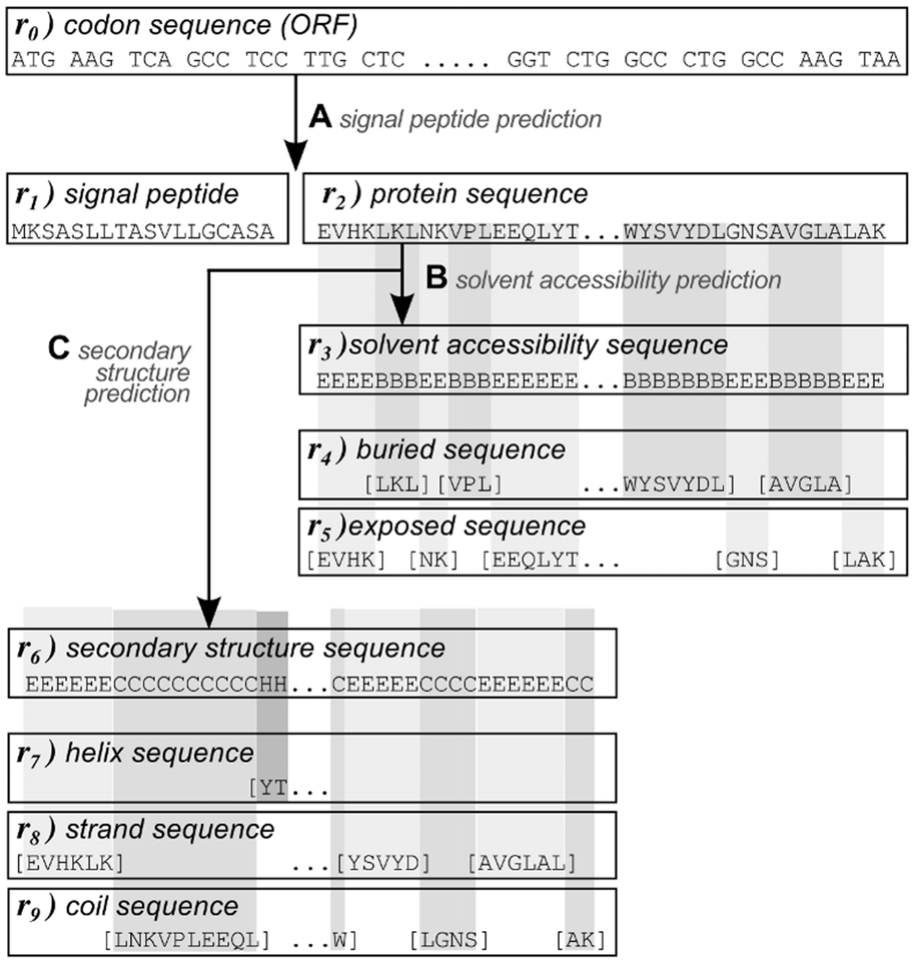
Different sequence-based protein representations. The different shades of gray denote predicted buried (B) and exposed (E) regions in case of the the solvent accessibility, and predicted helix (H), strand (E), and random coil (C) region in case of the secondary structure.

Figure 1 shows the ten different sequences that were used to represent a protein: 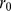) the ORF codon sequence, using a 64 letter codon alphabet; 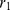) the N-terminal signal peptide sequence; 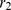) the mature protein sequence (excluding the signal peptide); 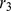) the predicted solvent accessibility sequence, using B for buried and E for exposed; 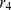) the parts of the mature protein sequence predicted to be buried, and 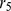) to be exposed, both using the 20 letter amino acid alphabet; 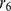) the predicted secondary structure sequence, using H for 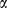-helix, E for 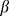-strand, and C for random coil; 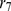) the parts of the mature protein sequence predicted to be in a helix structure; 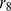) in a strand structure; and 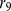) in a random coil region, all three using the 20 letter amino acid alphabet.

We used randomized versions of the different structural sequences: 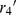) randomized buried sequence, 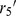) randomized exposed sequence, 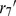) randomized helix sequence, 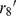) randomized strand sequence, and 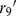) randomized coil sequence, to test whether their actual amino acid content or just their length is predictive. For example, if for a given protein 50 residues are predicted to be in a helix structure, i.e. the helix sequence has length 50, a randomized helix sequence is constructed by selecting 50 residues from the entire protein sequence at random.

### Structural Predictions

SignalP 3.0 [21] was used to predict N-terminal signal peptide presence and signal peptide cleavage site. From the neural network output, we used the default D-value threshold (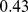) to decide if a protein contains a signal peptide and used the predicted signal peptide cleavage site to split a protein sequence into a signal peptide part and a mature protein sequence part (Figure 1A). NetSurfP 1.0 [25] was used to predict structural location (either buried or exposed) of each amino acid in a mature protein sequence (Figure 1B). PsiPred 3.21 [26] was used to predict secondary structure of the mature protein sequence, using UniRef90 as a database (Figure 1C).

### Classification

A linear support vector machine (LIBSVM [27]) was used for classification [28], in which the prediction 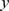 is a weighted combination of kernels 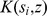 between the training objects 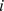 and a test object 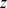:
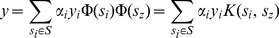
(1)


For each object (protein) 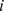, 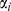 is the weight assigned to the object as obtained from the trained classifier (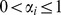 if the object is a support vector, 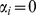 otherwise), 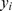 the class label (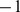 or 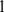), 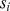 the sequence of protein i, and 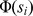 a mapping from sequence to feature space. The SVM is trained by optimizing a quadratic programming problem:
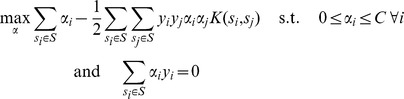
(2)


The parameter 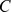, controlling the trade-off between training error and classifier complexity, was optimized using a simple grid search over 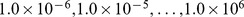. Classifier performance on a data set was estimated by running a double 10-fold cross-validation (CV) loop, in which 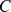 was optimized in an inner CV-loop on the training set. As performance measure we used the area under the receiver-operator characteristic curve (auroc) [29]. Classifier performance is defined as the average auroc over the CV-loops. When separate training and test sets are used, a classifier was trained on the first data set, optimizing 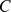 in a 10-fold CV-loop, and tested on the second data set, again using the auroc as performance measure.

In the cross-validation error estimation procedure, a predictor is repeatedly trained on 90% of the data set and tested on the remaining 10% of the data set. If features derived from a training set that are important for discriminating between the positive and negative class also yield good performance on the test set, then these features apparently allow good generalization. In this sense, a good CV performance can be interpreted as an *in silico* validation of the features found.

### Classifier Interpretation and Comparison

For a given set of sequences 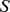, the feature weight vector 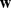 from a trained SVM classifier was obtained using:
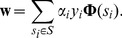
(3)


Classifiers were compared by taking the correlation between 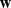 of both trained classifiers. A high correlation indicates a high similarity between the classifiers, both assigning similar weights to the same features.

### Feature Sets

We derived distinct sets of sequence-based features, 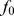
–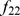, which will be described below. A visualization of feature matrices 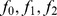, and 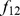 for 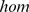 and 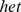 are given in Supplementary Figures S1–S8. Features 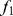
–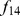 were used in an inner product kernel (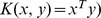; for features 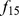
–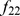 we used a spectrum kernel (see below).

#### Composition-based features

(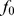 – 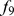) The composition of sequences 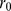 – 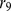 (Figure 1). For a sequence 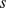 on alphabet 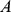, the composition 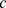 is defined as:
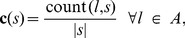
(4)in which count (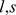) is a function that counts the number of occurrences of letter 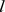 in sequence 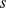, and 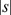 is the length of the sequence. The size of the feature vector 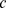 depends on the size of alphabet 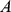, e.g. the composition of the codon sequence 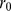 results in a feature vector of length 64 and the composition of the protein sequence 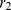 results in a feature vector of length 20. This means that 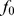 and 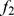 consist of 64 and 20 features respectively. 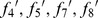 and 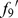 are the compositional features of the randomized sequences 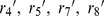 and 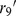



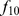
) Predefined amino acid cluster composition of 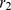 using the 11 predefined clusters in Table 1. The clusters are based on those defined in [30]. (Note: In this clustering it sometimes occurs that an amino acid is both inside and outside a cluster, based on its state; e.g. a free cysteine is in the polar cluster, while a cysteine that forms a disulfide bridge is outside the polar cluster. Without structural data, amino acid states are unknown. We therefore removed an amino acid from the cluster if it also resides somewhere outside that cluster, i.e. cysteine is not considered to be part of the polar cluster.) For a sequence 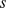 and clusters 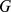, the cluster composition vector 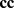 is defined as:
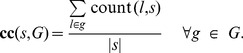
(5)


**Table 1:** Predefined amino acid clusters.

cluster	amino acids
small	V, C, A, G, T, P, S, D, N
polar uncharged	S, W, N, Q, T, Y
aromatic	F, Y, W, H
acidic	D, E
charged	H, K, R, E, D
basic	K, R, H
hydrophobic	I, L, V, M, F, Y, W, H, C, A, T, K
tiny	A, G, S
nonpolar	A, V, L, I, M, G, F, P
aliphatic	I, L, V
polar	Y, W, H, K, R, D, E, T, S, N, Q


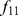
) Optimized amino acid cluster composition of the protein sequence (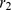) using clusters that are optimized for our data set using the method described in the next section (Amino acid clustering).

#### Sequence-derived features


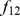
) Using 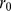, codon usage was calculated for the 59 codons that non-uniquely encode for an amino acid. Codon usage is defined as the codon count divided by the amino acid count of the amino acid it encodes for.


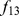
) Four other sequence-derived features: the signal peptide length, the protein sequence length, the codon adaptation index [31] that was calculated using a codon usage index derived from all *A. niger* genes, and the isoelectric point. The last two values were calculated using the codon sequence (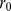) and the protein sequence (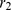) respectively, both using the Biopython software package [32].

#### Selected features


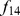
) A two-sample 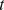-test (python SciPy package [33]), was applied to a set of 124 features, combining the features from feature sets 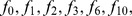 and 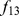. Features with a 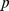-value 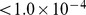 were selected for forward feature selection, 36 and 33 features for 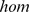 and 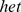 respectively (Supplementary Table S4).

In a 10-fold cross-validation loop, forward feature selection was applied on the training set. Features were added one by one, based on their prediction performance as determined using a second inner 10-fold CV-loop, until prediction performance starts to drop. To reduce calculation time, parameter 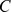 was not optimized but based on observations fixed to 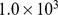 and 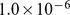 for 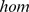 and 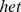, respectively. The selected features per CV-loop for both 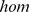 and 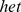 are given in Supplementary Table S5.

#### Pattern-based features


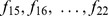
) We employed spectrum kernels [34], which define similarities between sequences based on fixed-length subsequence (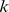-mer) counts, as implemented by Shogun [35] to search for predictive patterns. We calculated 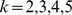 spectrum kernels using 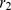 (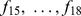) and 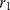 (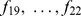).

### Amino Acid Clustering

We developed a method that forms amino acid clusters using our data sets, thereby constructing new features optimized for our data. A cluster is defined as a set of one or more amino acids. For the resulting clusters, each amino acid can be in one cluster only, not every amino acid needs to be in a cluster.

The method starts with selecting the best performing amino acid, i.e. the amino acid that, when used as the only feature, provides the best classification performance, the same as in forward feature selection. For example, if the fraction of lysine in a protein provides the best separation between the positive and negative class, this amino acid will start the first cluster. In the next iteration, for the remaining 19 amino acids, classification performance is tested for two cases: 1) with the amino acid added as new cluster and 2) with the amino acid added to the existing cluster. In case of the example, when adding alanine, classification performance is tested both using the fraction of lysine and the fraction of alanine as two separate features, and using the sum of the fractions of lysine and alanine as a single feature. The case that provides the best classification performance is selected. In the next iteration, with 18 amino acids remaining, the same procedure is applied. This iteration cycle is proceeded until there are no more amino acids left. Finally, the overall best performing clusters are the output of the method. Consequently, it might happen that some amino acids will not be selected at all.

This procedure is implemented in a 10-fold CV-loop, obtaining the best performing clusters on the training set and using them as cluster composition features on the test set. The selection protocol is applied in an inner CV-loop to avoid biases towards the training data. The obtained clusters per CV-loop are given in Supplementary Table S6.

### Statistical Pattern Discovery

The statistical motif finding approach MEME [36] was used to find patterns (described as position-dependent letter-probability matrix) that occur once in every sequence (oops mode) of a data set. Discriminative motif discovery was performed using the successful secreted proteins as input with the unsuccessful secreted proteins as negative sequences and vice versa. This was done for both 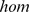 and 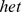. The minimal and maximal motif lengths were set to 2 and 15 respectively.

## Results

### Sequence Data is Predictive for High-level Protein Production

To test if the sequence data is informative, we used it to predict successful high-level protein production. Classifiers were built using an extensive set of sequence-based features. Performance results (auroc) of 10-fold CV experiments on both 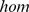 and 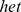 are shown in Table 2, 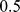 indicating random prediction and 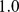 perfect prediction. Best classification performances of 0.85 and 0.75 auroc respectively (boldface in Table 2) show that sequence data is predictive. As additional support, classifier outcome for the *A. niger* proteome (Supplementary Figure S11) shows an expected result, predicting successful high-level production for only a fraction of the proteome.

**Table 2:** Prediction performance scores (auroc).

features	*hom*→*hom*	*het*→*het*	
**Composition-based features**			
*f*_0_	**0.85**	0.70	*ORF codon composition*
*f*_1_	0.66	0.51	*signal peptide AA composition*
*f*_2_	0.83	0.70	*mature protein AA composition*
*f*_3_	0.68	0.51	*buried-exposed composition*
*f*_4_ (*f*_4_′)	0.80 (0.80)	0.65 (0.64)	*buried AA composition*
*f*_5_ (*f*_5_′)	0.82 (0.78)	0.64 (0.65)	*exposed AA composition*
*f*_6_	0.62	0.57	*helix-strand-coil composition*
*f*_7_ (*f*_7_′)	0.68 (0.70)	0.60 (0.57)	*helix AA composition*
*f*_8_ (*f*_8_′)	0.70 (0.72)	0.61 (0.57)	*strand AA composition*
*f*_9_ (*f*_9_′)	0.80 (0.80)	0.65 (0.65)	*coil AA composition*
*f*_10_	0.80	0.63	*AA clusters composition*
*f*_11_	0.83	0.67	*optimized AA clusters comp.*
**Sequence-derived features**			
*f*_12_	0.64	0.54	*codon usage*
**Selected features**			
*f*_14_	0.84	**0.75**	*feature selection*
**Pattern-based features**			
*f*_15_	0.82	0.63	*2-mer counts protein*
*f*_16_	0.77	0.61	*3-mer counts protein*
*f*_17_	0.68	0.60	*4-mer counts protein*
*f*_18_	0.57	0.47	*5-mer counts protein*
*f*_19_	0.63	0.54	*2-mer counts signal peptide*
*f*_20_	0.59	0.52	*3-mer counts signal peptide*
*f*_21_	0.54	0.51	*4-mer counts signal peptide*
*f*_22_	0.56	0.50	*5-mer counts signal peptide*

Considering the composition-based features, similar results were observed for the codon sequence (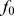) and the protein sequence (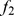), which is expected because of the relation between the two sequences. For 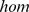, high performance using protein sequences is in line with results of our previous work [37]. Similarly, results of other previous work, regarding only protein localization and not production rate, reported different amino acid compositions for intra- and extracellular proteins [38], [39]. Although the codon sequence shows a slightly higher score for 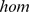, it does not significantly outperform the protein sequence (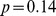 for a paired 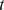-test on the test scores of the 10 CV-loops).

The predictive power of the amino acid composition of the signal peptide (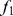) proves to be limited, clearly outperformed by both the codon and protein sequence. More advanced methods, taking into account letter/pattern location [40], [41], did not improve prediction results (results not shown).

### Similar Characteristics are Important for Both Data Sets

**Figure 2:**
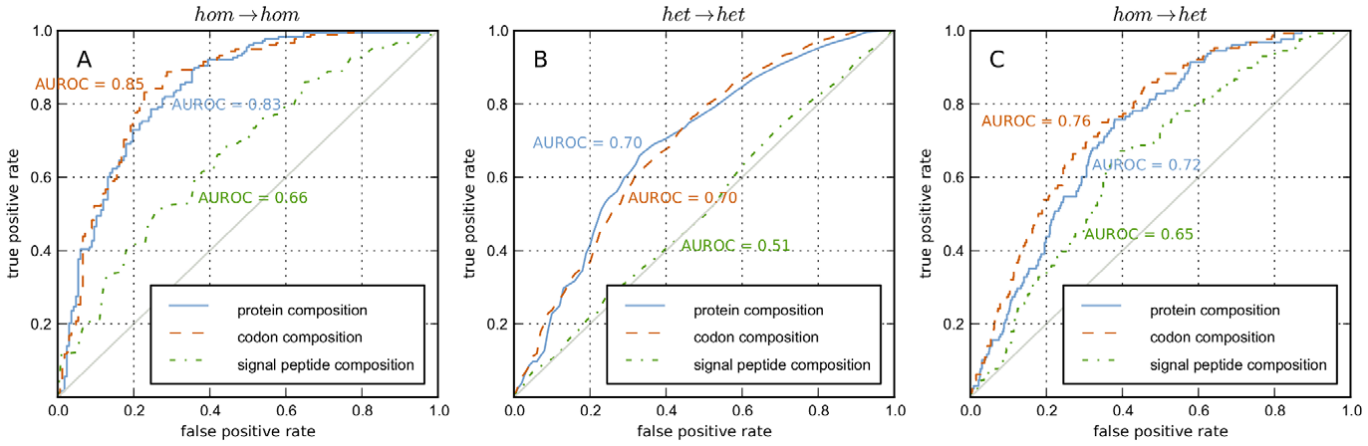
Classification performances. ROC-curves of composition-based classifiers using the codon sequence (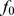), the signal peptide sequence (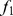), and the protein sequence (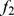). Performances are shown for classifiers **A)** trained and tested on 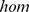, **B)** trained and tested on 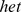, and **C)** trained on 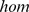 and tested on 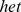.

Figure 2 shows the ROC-curves of the composition-based classifiers discussed thus far. Figure 2A and Figure 2B show the average result of a 10-fold CV-loop on 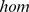 and 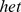 respectively. Figure 2C shows the result of a classifier trained on 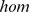 and tested on 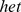. Remarkably, this shows similar results as the classifiers trained on 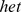, suggesting that the homologous classifier generalizes well to predict high-level production for 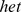. In fact, the classifiers trained on 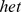 performed even slightly worse. This might be due to the fact that this data set is too heterogeneous, originating from 14 different species, which makes it harder to build a generic classifier and may have caused over-fitting in the CV-loops.

**Figure 3:**
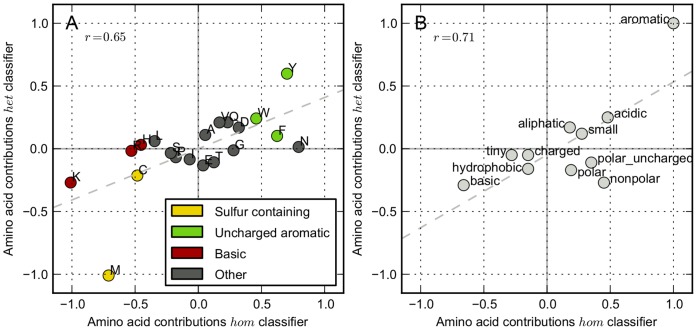
Comparing *hom* and *het* classifiers. Amino acid contributions obtained from 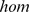 and 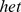 trained classifiers are the 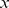
- and 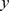-values respectively, the correlation is denoted by 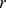. Contributions are normalized per classifier (axis): each contribution is divided by the maximum absolute contribution. The plots show the contributions obtained from classifiers trained using **A)** the protein amino acid composition (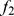) and **B)** the predefined amino acid cluster composition (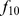).

The good generalization of the 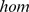 classifier on the 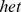 data set suggests that classifiers trained on 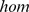 and 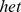 are similar, i.e. perform their predictions based on the same sequence characteristics. The correlation of 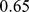 in Figure 3 shows that this is indeed the case. The figure shows the contribution of each amino acid as obtained from the 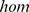 and 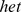 classifier, both trained using the protein amino acid composition (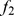). Positive values indicate contribution to successful high-level production and negative values indicate contribution to unsuccessful high-level production.

For both 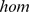 and 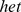, a remarkable positive and negative contribution of respectively tyrosine (Y) and methionine (M) is apparent. For 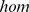, also asparagine (N) and lysine (K) show an outstanding positive and negative contribution respectively. Considering amino acid properties, it is observed that the basic and the sulfur-containing amino acids have a negative contribution whereas the (uncharged) aromatic amino acids have a positive contribution.

Besides comparing the amino acid contributions of the 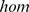 and 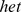 classifier, we also compared them to amino acid synthesis costs as defined in [42]. With the exception of the aromatic amino acids, a negative correlation is shown between the 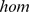 contributions and the amino acid costs (Supplementary Figures S9–S10), suggesting a preference for “cheap” amino acids for high-level secretion.

### Basic and Aromatic Amino Acids are Predictive

From a structural and functional perspective, it is often more useful to look at the physicochemical properties of an amino acid, rather than looking at the 20 amino acids as different entities. Therefore, based on physicochemical properties [30], we defined 11 predefined amino acid clusters (Table 1), and used these as features (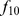). In this case, a correlation of 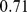 was observed between the 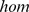 and 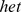 classifier (Figure 3B). The aromatic amino acids have a high contribution to high-level production, which, looking back at Figure 3A, is similar to the amino acid contributions, except for the positively charged histidine (H). A negative contribution is observed for the basic amino acids, also consistent with the observations in Figure 3A.

**Figure 4:**
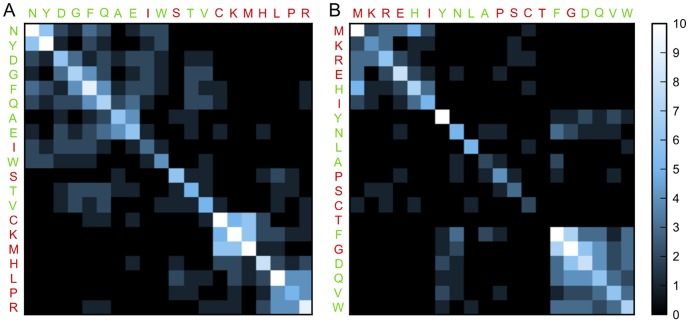
Best performing amino acid clusters. The heat maps show the combined result of the best performing clusters obtained in 10 CV-loops for both 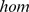 (**A**) and 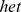 (**B**). The values on the diagonals denote how often an amino acid ended up in a cluster (due to selecting the optimal clusters, amino acids might not be selected at all). The colors on the non-diagonal places denote how often two amino acids ended up in the same cluster. Complete linkage hierarchical clustering was used to cluster the heat map, using the euclidean distance as distance measure. The color of the amino acid letters indicates if the amino acid has a positive (green) or negative (red) contribution in Figure 3A.

Since it is unclear which amino acid clusters are suitable for what problem, we developed a novel method that uses the data set to construct clusters. The best performing clusters (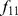) obtained in ten CV-loops are jointly shown as a heat map in Figure 4. The non-diagonal values show the number of times that two amino acids were found in the same cluster. The diagonal values show how often an amino acid was found in any cluster.

The diagonal values correspond to the results observed in Figure 3A: highly contributing amino acids were often found in a cluster. For 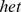, noteworthy exceptions are phenylalanine (F) and glycine (G), both of which always ended up in a cluster despite their low contribution.

The non-diagonal values also match the results in Figure 3A. As can be observed, amino acids with a positive contribution (green letters) and amino acids with a negative contribution (red letters) often form clusters, whereas amino acids with contradicting contributions rarely do. The occurrence of only few light cells show that not many amino acids consistently form the same cluster. Only clusters with phenylalanine (F), glycine (G), aspartic acid (D), and glutamine (Q) occur relatively often in both data sets, but those do not share an obvious physicochemical property. Despite the high contributions observed for the aromatic amino acids in Figure 3B, clustering of these amino acids occurred only a few times.

### Structural Subsequences have Limited Information

The secondary structure composition (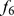) shows to be little predictive, with an auroc of 0.62 and 0.57 for 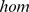 and 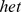 respectively. Results using the amino acid composition of the helix (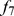), strand (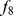), and coil sequence (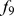) suggest that the coil sequence is more informative than the helix and strand sequence, however, a similar result was obtained using a randomized version of the sequence (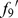, score between brackets in Table 2). This indicates that the coil sequence, although it provides higher classification performance, is not more informative than the helix and strand sequence. The better performance can be explained by the length of the sequence, proteins are on average composed of 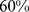 coil, 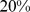 helix, and 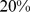 strand.

Considering the solvent accessibility, the distribution of buried and exposed amino acids (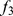) is only predictive for 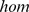 (auroc 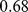). The buried amino acids showed a positive contribution to high-level production (data not shown). Results using the amino acid composition of the buried (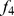) and exposed sequence (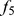), separately, are similar to the randomized buried (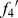) and randomized exposed sequence (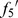), indicating that neither of the two sequences is more informative than a randomly selected sequence of the same length.

### Best Performance with Only Few Features

Thus far, all discussed classifiers were trained on a relatively small set of related features. Combining all features results in a large feature set which complicates both classification and interpretation. To resolve this, we used a forward feature selection protocol similar to the one used in previous work [37].

**Figure 5:**
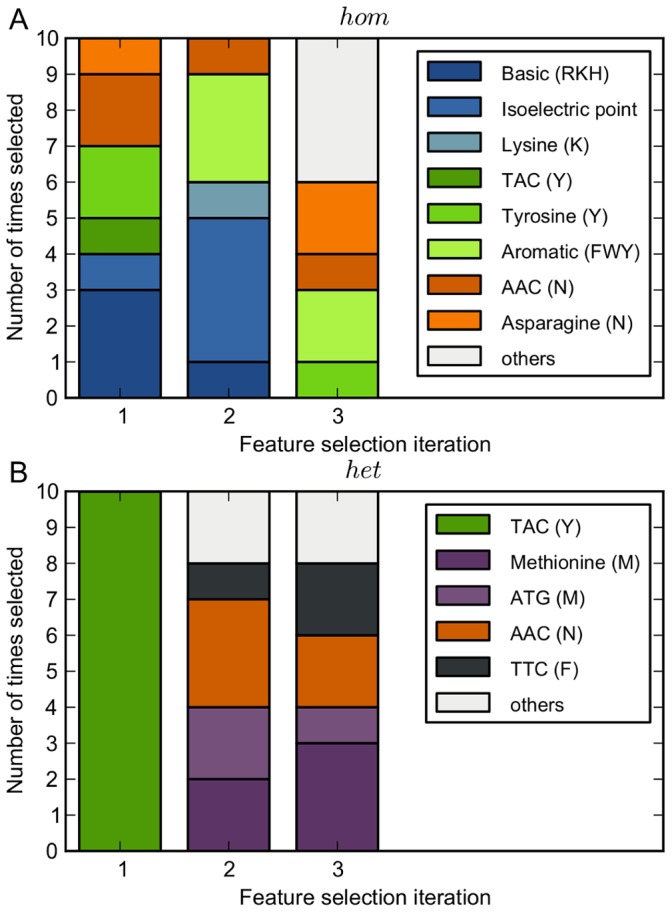
Feature selection. For the first three feature selection iterations (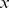-axis), the bar plot shows how often features were selected in the 10 CV-loops for both 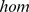 (**A**) and 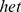 (**B**). Features with a different shade of the same color are correlated (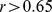). The letters between brackets in the legend are amino acids that denote either which amino acids are in the cluster, e.g. the basic cluster contains amino acids R, K, and, H, or for which amino acid a codon encodes, e.g. codon TAC encodes for Y.

A classification performance of 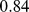 auroc was obtained for 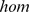 (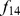 in Table 2), similar to the results obtained using the protein’s amino acid or codon composition. Interpretation of the selected features shows a similar trend compared to the amino acid contributions observed in Figure 3A. As shown in Figure 5A, the first three selected features were almost always lysine (K), tyrosine (Y), and asparagine (N), or, as shown by a different shade of the same color, a correlated feature (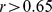).

For 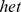, feature selection resulted in the best obtained prediction performance of 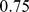 auroc (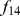). A relatively low number of features, on average six, were selected each CV-loop, most of which were codons. Remarkably, the codon TAC (Y) was consistently selected first (Figure 5B). Methionine (M and ATG) and the codons AAC (N) and TTC (F) were most often selected second and third.

The fact that codons are selected before amino acids suggests the importance of codon usage. However, taking codon usage as features provided an auroc of only 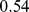 (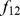). This could be due to the heterogeneous codon usage of the different organisms in 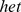. With an auroc of 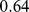, codon usage in 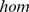 appeared to be a little predictive.

### A Role for N-glycosylation Motifs

Functional patterns, often called (short) linear motifs [43] (SLiMs), have been associated with protein targeting. The most well-known example is the C-terminal [HK]DEL motif that causes ER retention. Also a case with a secretion specific signal has been identified [44].

All proteins in our data sets contain a signal peptide, proteins with an ER-retention signal and proteins with predicted transmembrane regions are filtered out. Still, successful high-level production was observed for only half of the proteins in 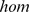. Unsuccessful high-level production could for example be caused by a low production rate or a high degradation rate, resulting in a too low concentration to detect on the gel (i.e. 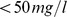). An alternative explanation could be the existence of additional retention or targeting signals. The statistical motif finding approach MEME [36] was used to search for such signals.

For 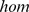, the pattern 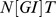, which matches the N-glycosylation pattern 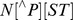, was found for successful high-level production. Instead of retention or targeting, this indicates importance of this post-translational modification. No other patterns related to either successful or unsuccessful high-level production were found, indicating the absence of additional generic targeting or retention signals.

SLiMs related to post-translational modifications can occur more than once in a sequence. Therefore we also searched for reoccurring patterns by building classifiers using fixed length pattern (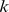-mer) counts as features. Using the signal peptide and the protein sequence, results for 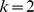 to 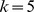 are shown in Table 2 (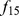
–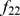). In general, classification performances rapidly drop with increasing pattern length, caused by an explosion of the number of possible 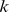-mers that results in sparse kernels [28] which are difficult to use for classification. Again, the N-glycosylation pattern was identified. Inspection of the 3-mer classifier trained on 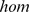 showed that six out of the seven 3-mers with the most positive contribution match the N-glycosylation pattern.

The N-glycosylation pattern is much more abundant in 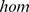 than in 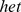, with on average 3.37 N-glycosylation patterns per protein for 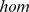 compared to an average of 1.42 per protein for 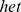. A clear difference is observed between the positive and negative proteins in 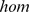, containing an average of 4.71 and 1.95 patterns respectively. Although much smaller, with an average of 1.72 and 1.36 patterns for the positive and negative class, respectively, 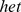 shows a difference as well, suggesting that the addition of N-glycosylation sites might be useful to improve heterologous secretion [45].

## Discussion

Using machine learning techniques, we explored which combinations of a large number of features best helps predicting successful high-level protein production in *A. niger*. The results show that composition-based features were most predictive, but that the exact representation – by codons, amino acids or amino acid clusters – has little influence. Taking into account predicted structural location of the amino acids did not further improve prediction results. Although all proteins have a signal peptide and the signal peptide is usually cleaved off in the ER [46], its sequence is still somewhat predictive. This suggests a role for the signal peptide in determining translocation efficiency, possibly due to a higher affinity to the SRP.

Classifiers trained on 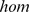 and 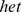 showed similar amino acid contributions, indicating that the properties found important for high-level production are generic in nature. The fact that poorer prediction performance was still obtained for 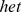 suggests that organism-specific properties may be important for high-level production. However, the heterogeneous nature of the 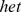 data and the resulting limited number of samples per donor organism hinder the identification of such properties using machine learning.

Feature selection on a larger set of features, including some derived from the sequence, confirmed that mainly composition-based features were selected in the first iterations. In fact, mainly codons and only a few amino acid features were selected for 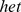. In the first three iterations, only codons were selected, implying room for production improvement by codon adaptation of heterologous proteins.

Among the composition-based features, a number of individual amino acids stood out as strongly contributing, either positively or negatively, to predicted high-level production:

- Tyrosine (Y), tryptophan (W) and phenylalanine (F) contribute positively. These aromatic amino acids are usually found in the protein core; their ability to form stacks can contribute to protein stability. A correlation between protein stability and secretion efficiency has been observed [47]–[49]. Moreover, improving secretion by increasing the protein stability is shown to be a successful strategy [50], [51]. It is hypothesized that proteins with a high stability more frequently escape from the ER quality control system, since they will more often be in the correctly folded state, which in general is the only state to leave the ER [48], [52].
- Asparagine (N) has a high positive contribution for 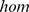. Since motif analysis showed the N-glycosylation pattern to be both predictive and abundant in 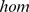 the contribution of asparagine could be related to this post-translational process in which a specific set of enzymes catalyzes the formation of N-linked glycans. Details are still unknown, but N-linked glycans are known to play an import role in protein folding and quality control [53]. Although N-linked glycosylation is not a prerequisite for secretion [54], there is ample evidence that introduction or modification of glycosylation sites can lead to improved secretion [45], [55], [56].
- Methionine (M) shows a strong negative contribution. The fact that it is a sulfur-containing amino acid, and that the other sulfur-containing amino acid, cysteine (C) also has a negative contribution, suggests a negative influence of sulfur-containing amino acids. Another explanation could be that methionine is encoded by the start codon ATG, which could slow down translation due to ribosome reinitiation on alternative start sites [57].
- Lysine (K) also has a strongly negative contribution, as do the other basic amino acids arginine (R) and histidine (H) for 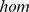. The positive charge, usually exposed at the protein surface, could facilitate binding to the negatively charged cell membrane, thereby preventing the protein to be filtered out, or could be related to protein thermostability due to charge-charge interactions on the protein surface [58].

In conclusion, we have exploited a large experimental dataset on production of proteins in *A. niger*, using both homologous and heterologous gene expression and employed machine learning algorithms to find combinations of features optimally predictive of presence or absence of high-level production. These features were all derived directly or indirectly from the protein sequences, and could be useful to improve industrial production rates of existing targets and to explore possibilities for new products. In future work, we intend to verify a number of the hypotheses provided here by engineering proteins to better reflect the features found to be related to high production rates.

## Supporting Information

Figure S1
**Shows the 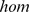 protein composition feature matrix (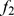).** The heat map visualizes the feature matrix with the features on the 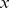-axis and the proteins on the 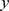-axis, the colors denote the feature value. Both the features (columns) and the proteins (rows) are clustered using complete linkage hierarchical clustering. The first bar to the right of heat map shows the protein labels, white for successful high-level production and gray for unsuccessful high-level production. The second bar to the right of the heat map shows from which donor organism the protein originates. In this case all proteins originate from *A*. niger, which is also the host organism.(PDF)

Figure S2
**Shows a heat map of the 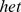 protein sequence composition feature matrix (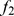), similar to Figure S1.**
(PDF)

Figure S3
**Shows a heat map of the 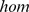 codon sequence composition feature matrix (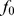), similar to Figure S1.**
(PDF)

Figure S4
**Shows a heat map of the 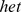 codon sequence composition feature matrix (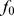), similar to Figure S1.**
(PDF)

Figure S5
**Shows a heat map of the 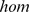 signal peptide composition feature matrix (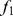), similar to Figure S1.**
(PDF)

Figure S6
**Shows a heat map of the 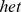 signal peptide composition feature matrix (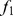), similar to Figure S1.**
(PDF)

Figure S7
**Shows a heat map of the 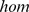 codon usage feature matrix (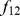), similar to Figure S1.**
(PDF)

Figure S8
**Shows a heat map of the 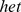 codon usage feature matrix (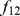), similar to Figure S1.**
(PDF)

Figure S9
**Shows the amino acid contributions of the 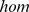 classifier (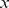-axis) versus amino acid costs (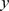-axis).** A correlation is observed for the non-aromatic amino acids, suggesting a preference for “cheap amino acids for high-level secretion.(PDF)

Figure S10
**Shows the amino acid contributions of the 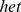 classifier (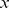-axis) versus amino acid costs** (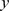-axis).(PDF)

Figure S11
**Shows the classifier outcomes of the 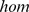 protein composition classifier.** **A)** The histogram shows the classifier outcomes for the 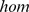 data set with the negatively labeled proteins in red and the positively labeled proteins in green. Note that the classifier is trained using the same data set. **B)** The histogram shows the classifier outcomes for the *A. niger* proteome in light grey. The subset of the proteome that contains a predicted signal peptide (SignalP 3.0) is shown in dark grey.(PDF)

Table S1
**Contains the labeled data set with** ***Aspergillus niger*** **proteins (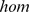) that were tested for successful high-level production and secretion.** Labels pos and neg indicate successful and unsuccessful high-rate production respectively.(XLS)

Table S2
**Contains the names and abbreviations of the 14 fungal donor organisms for which there are proteins in the heterologous data set (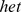).**
(TXT)

Table S3
**Contains the total number of proteins and the number of successfull and unsuccessfull proteins in both 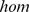 and 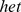, and per organism in 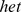.**
(XLS)

Table S4
**Contains the 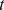-values and corresponding 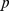-values as obtained from a two-sample 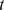-test on the 124 features that were used for feature selection.** For both 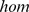 and 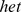, the features are ordered by the absolute 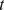-value.(XLS)

Table S5
**Contains the selected features in each CV-loop with forward feature selection on both 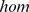 and 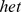.**
(XLS)

Table S6
**Contains the best performing amino acid clusters in each CV-loop obtained with the amino acid clustering procedure on both 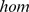 and 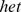.**
(TXT)
