# Supplementary figures and images for: Exploring Sequence Characteristics Related to High-Level Production of Secreted Proteins in Aspergillus niger
Source: PLoS One. 2012 Oct 1;7(10):e45869. doi: 10.1371/journal.pone.0045869 (PMC3462195; doi:10.1371/journal.pone.0045869)

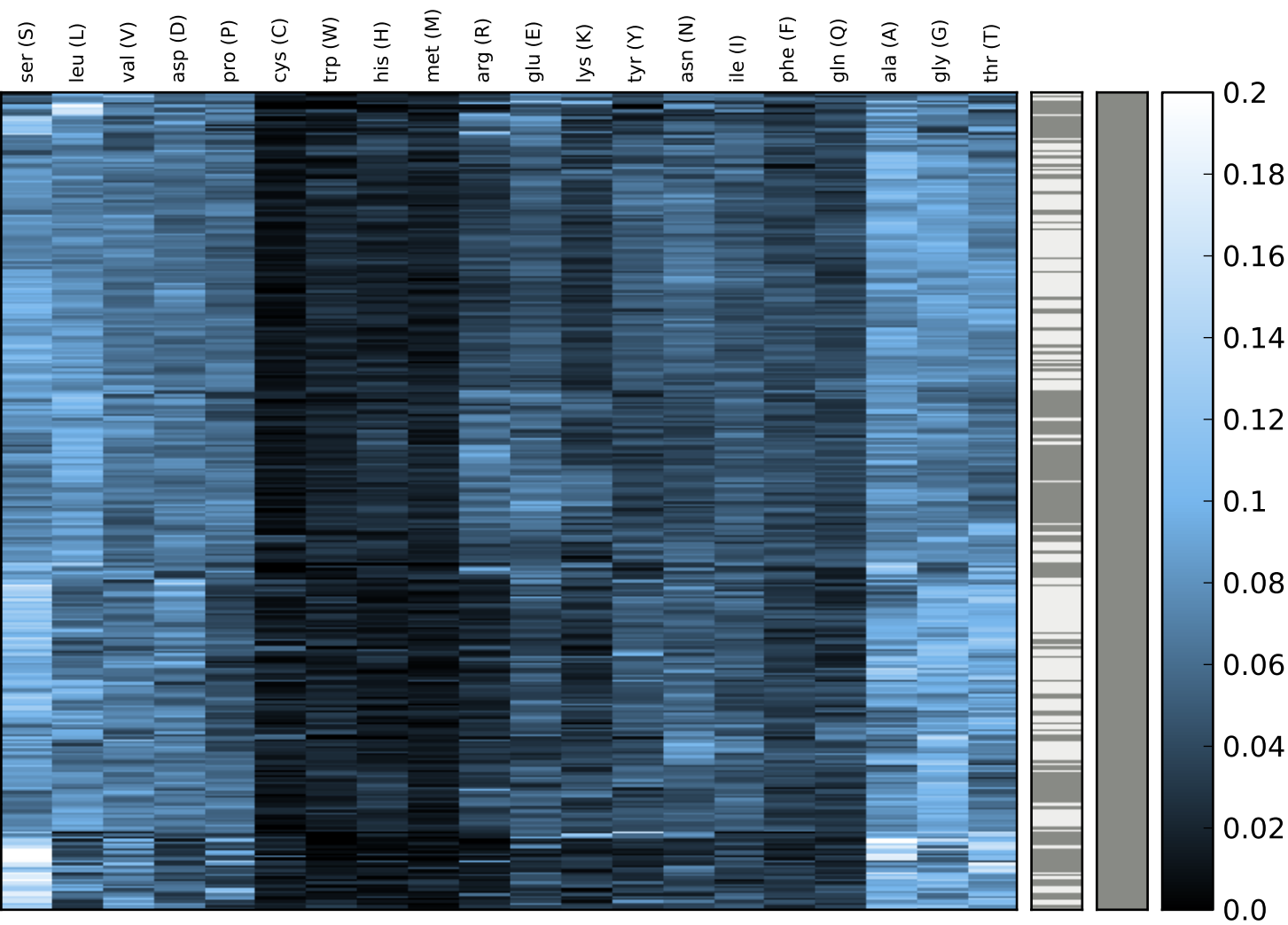

Supplement: Figure S1 — Shows the protein composition feature matrix (). The heat map visualizes the feature matrix with the features on the -axis and the proteins on the -axis, the colors denote the feature value. Both the features (columns) and the proteins (rows) are clustered using complete linkage hierarchical clustering. The first bar to the right of heat map shows the protein labels, white for successful high-level production and gray for unsuccessful high-level production. The second bar to the right of the heat map shows from which donor organism the protein originates. In this case all proteins originate from A. niger, which is also the host organism. (PDF) [file pone.0045869.s001.pdf]

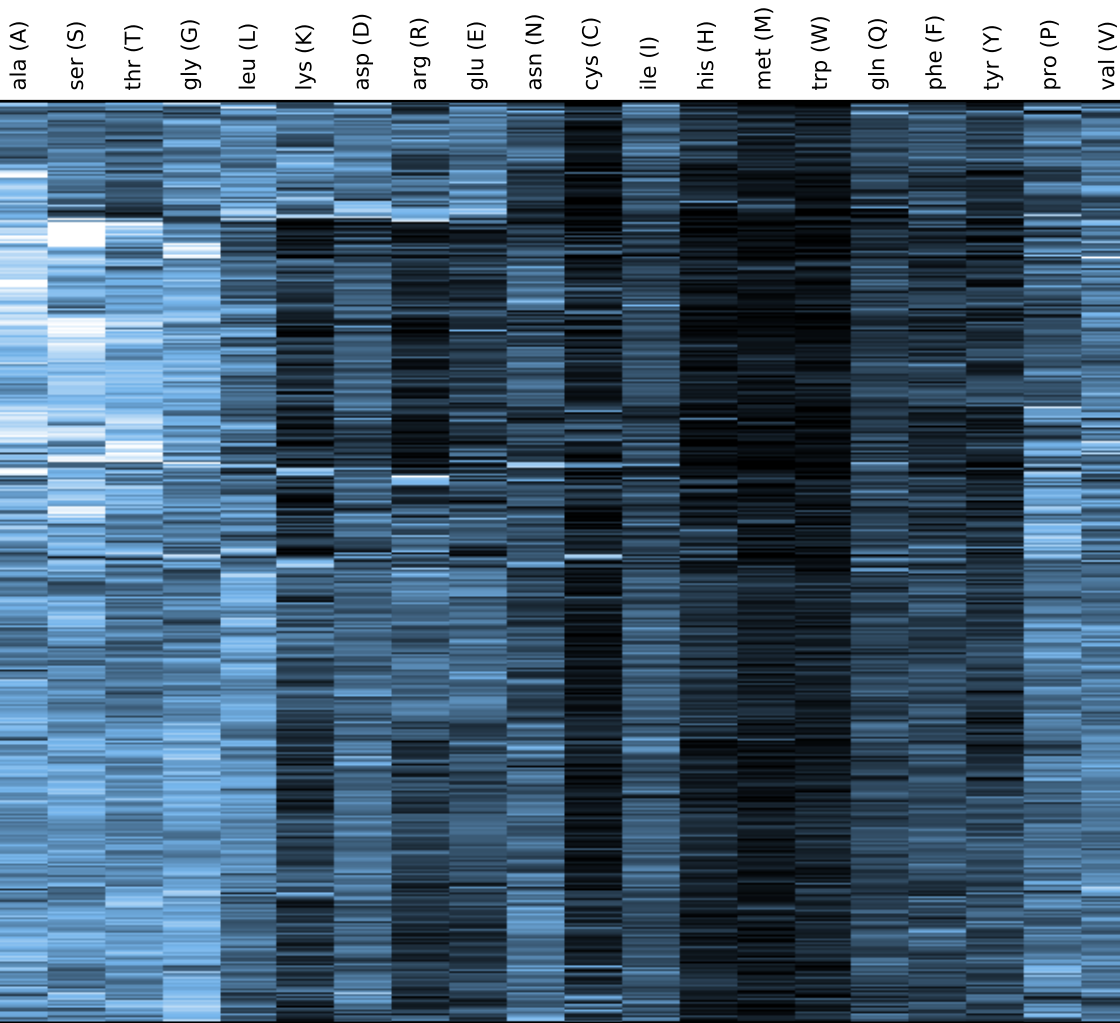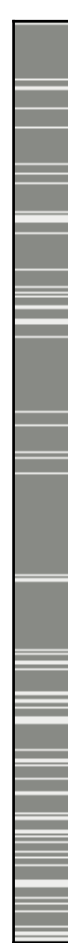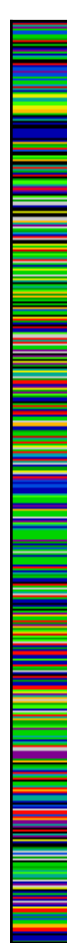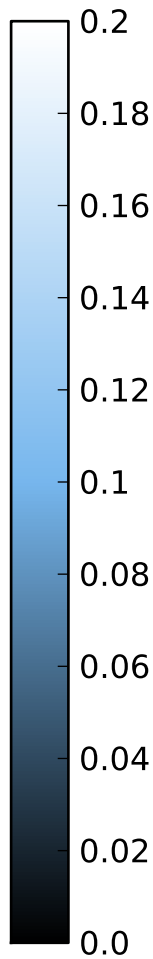

Supplement: Figure S2 — Shows a heat map of the protein sequence composition feature matrix (), similar to Figure S1. (PDF) [file pone.0045869.s002.pdf]

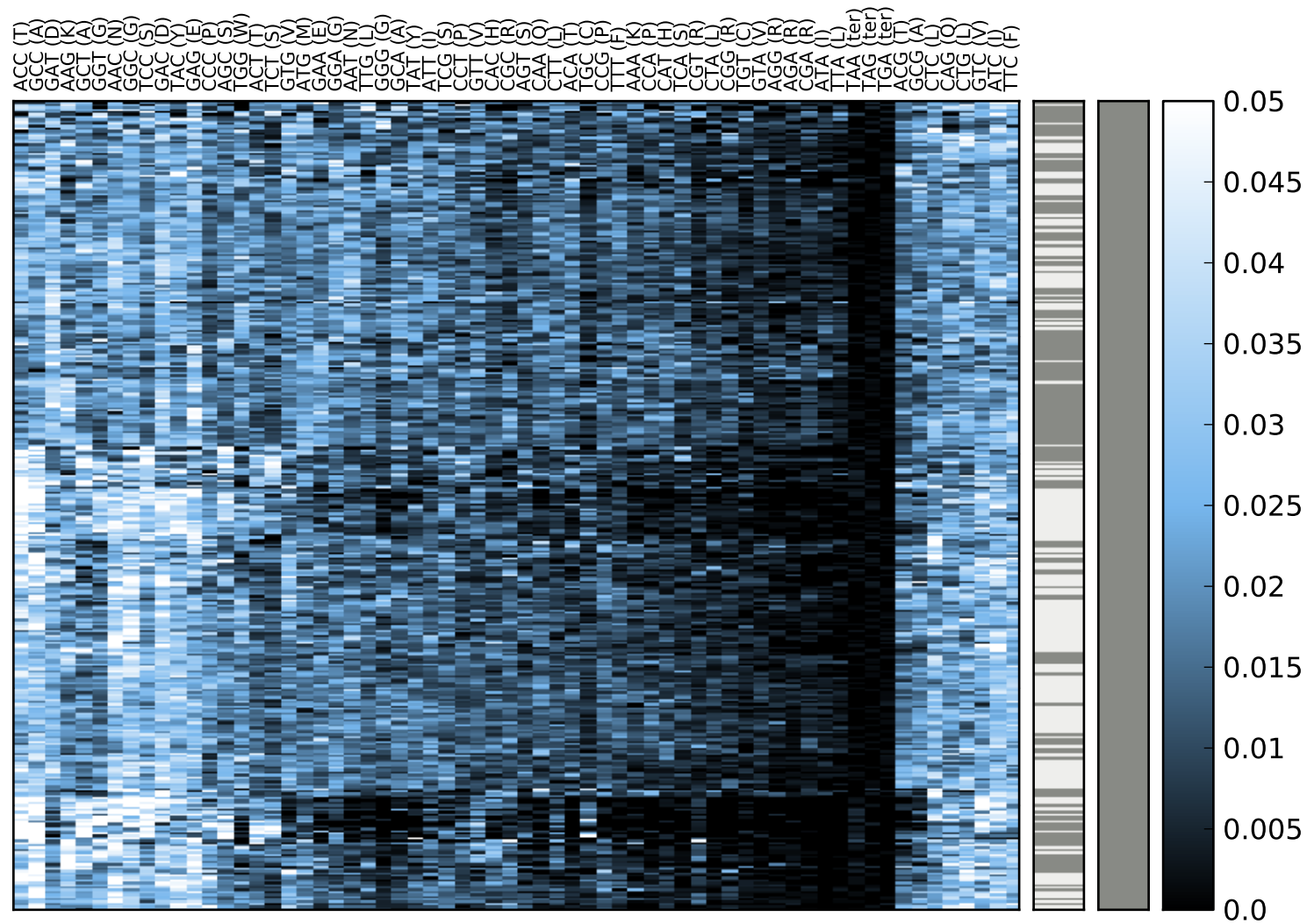

Supplement: Figure S3 — Shows a heat map of the codon sequence composition feature matrix (), similar to Figure S1. (PDF) [file pone.0045869.s003.pdf]

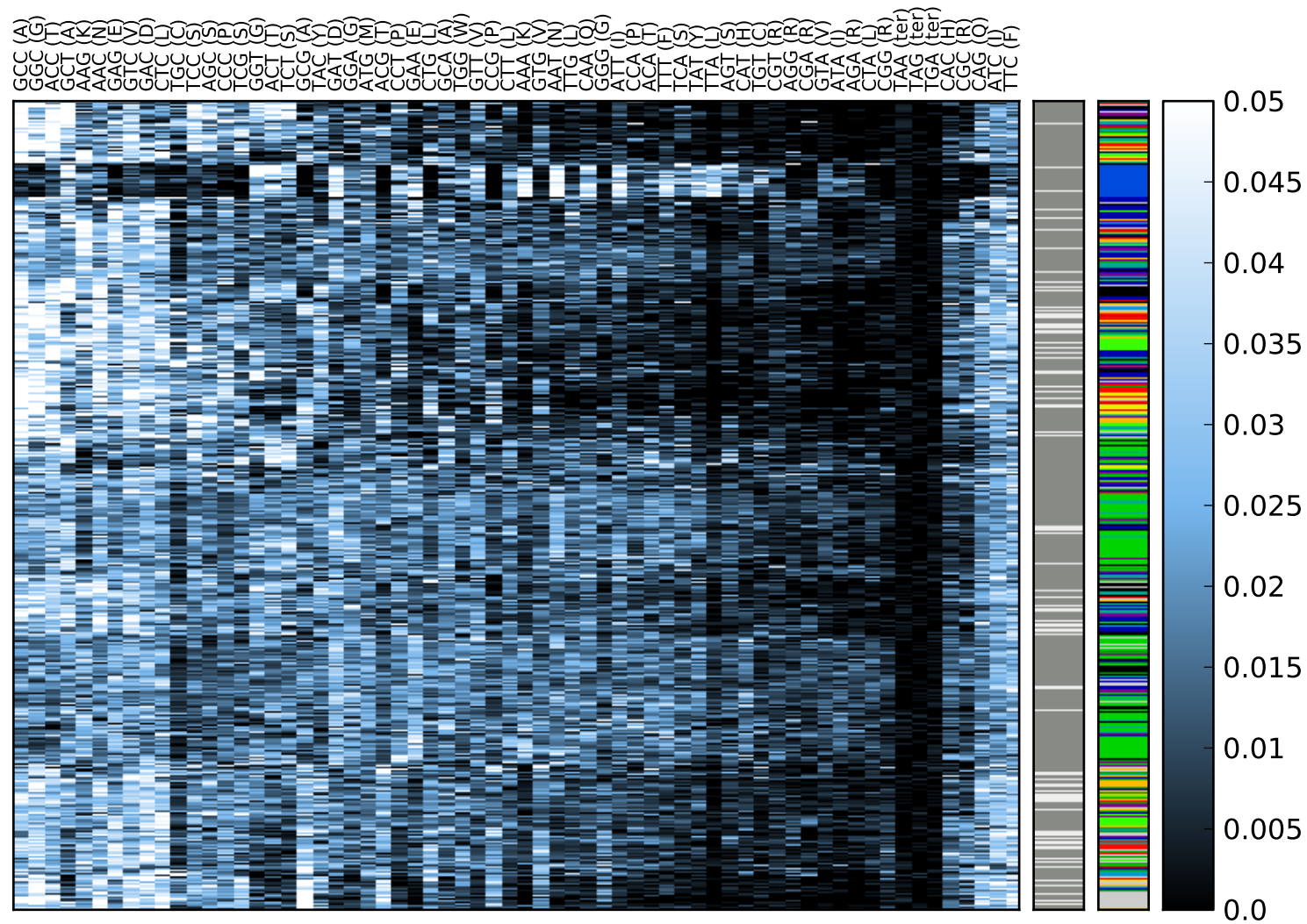

Supplement: Figure S4 — Shows a heat map of the codon sequence composition feature matrix (), similar to Figure S1. (PDF) [file pone.0045869.s004.pdf]

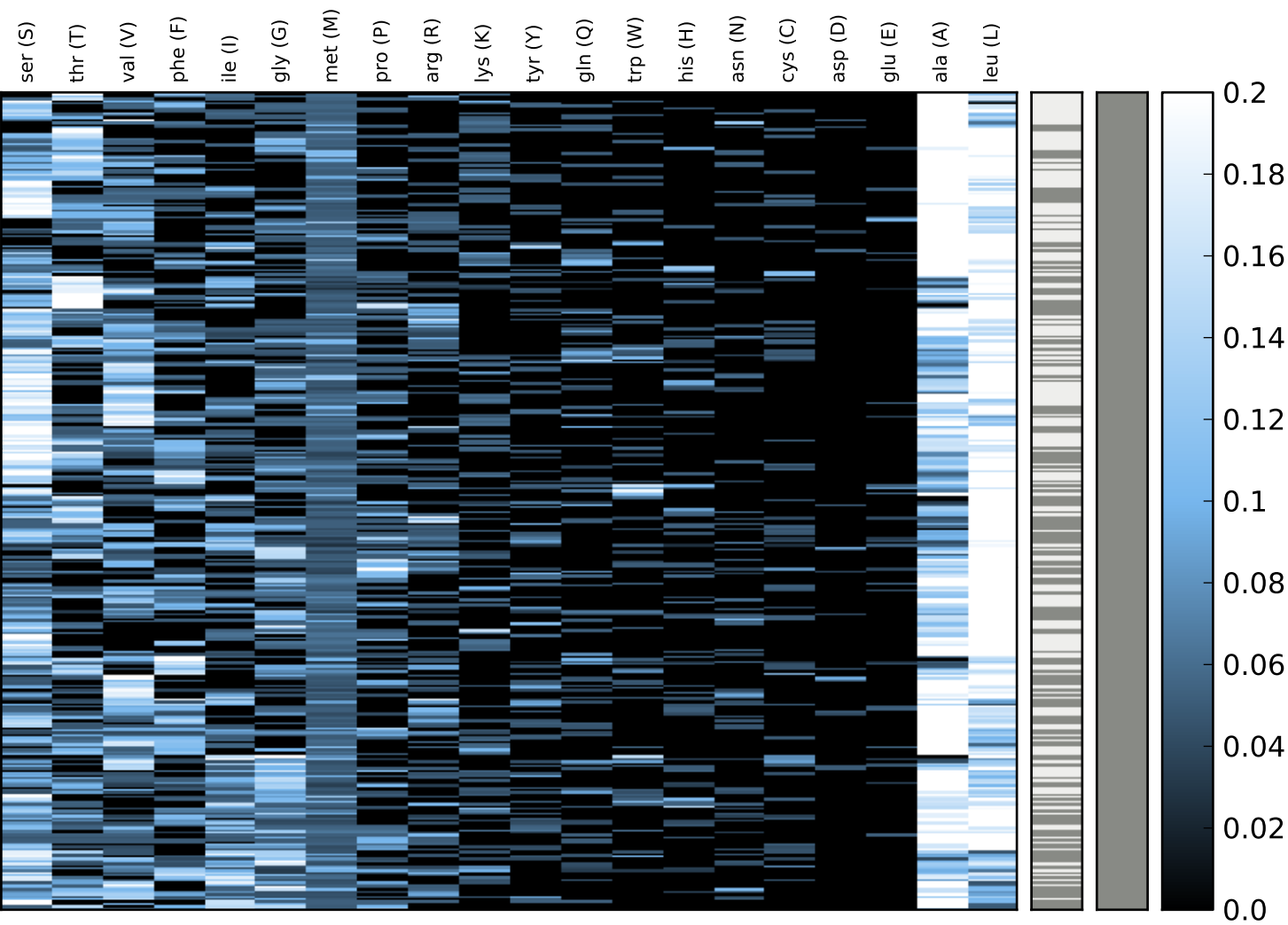

Supplement: Figure S5 — Shows a heat map of the signal peptide composition feature matrix (), similar to Figure S1. (PDF) [file pone.0045869.s005.pdf]

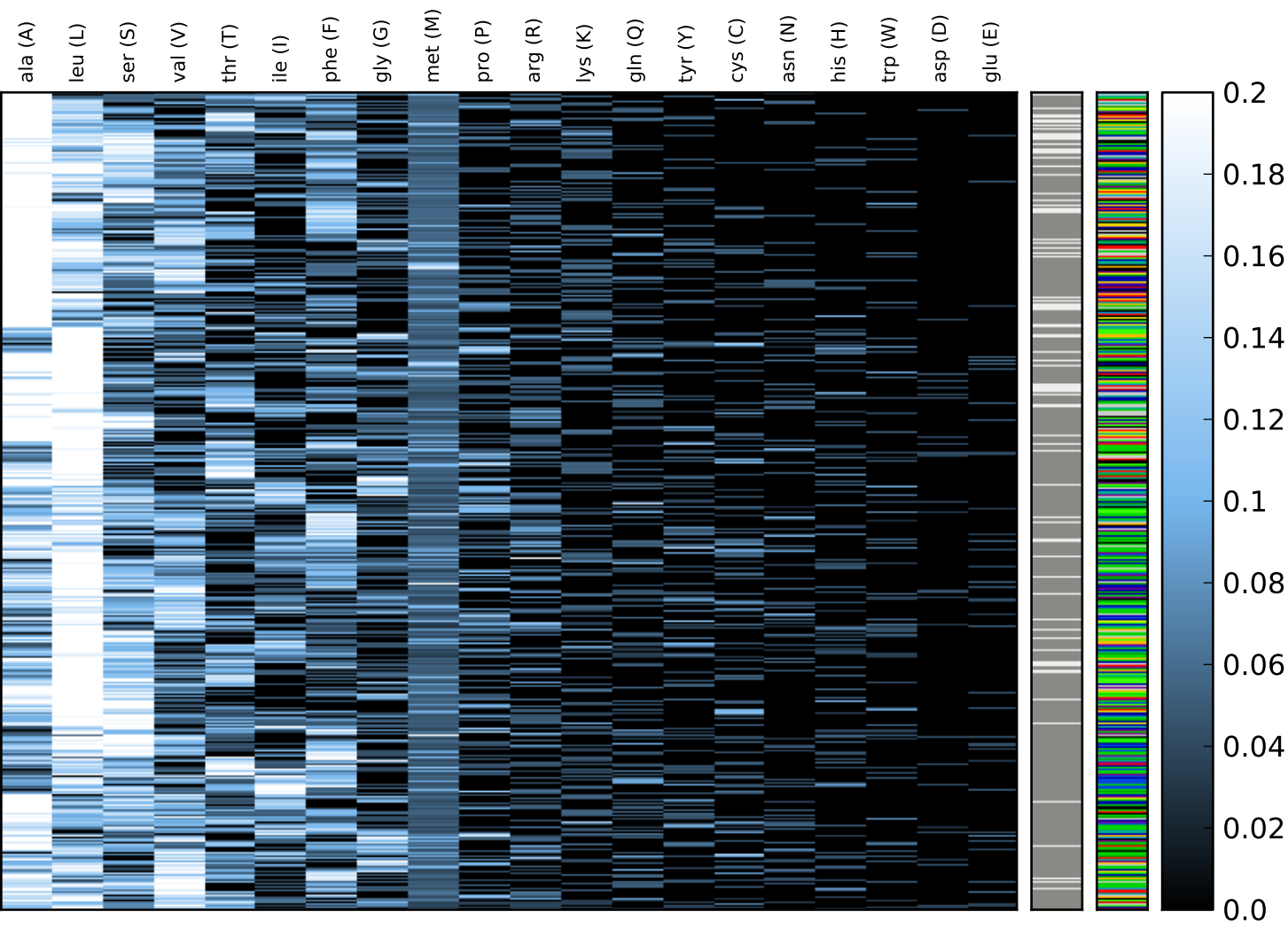

Supplement: Figure S6 — Shows a heat map of the signal peptide composition feature matrix (), similar to Figure S1. (PDF) [file pone.0045869.s006.pdf]

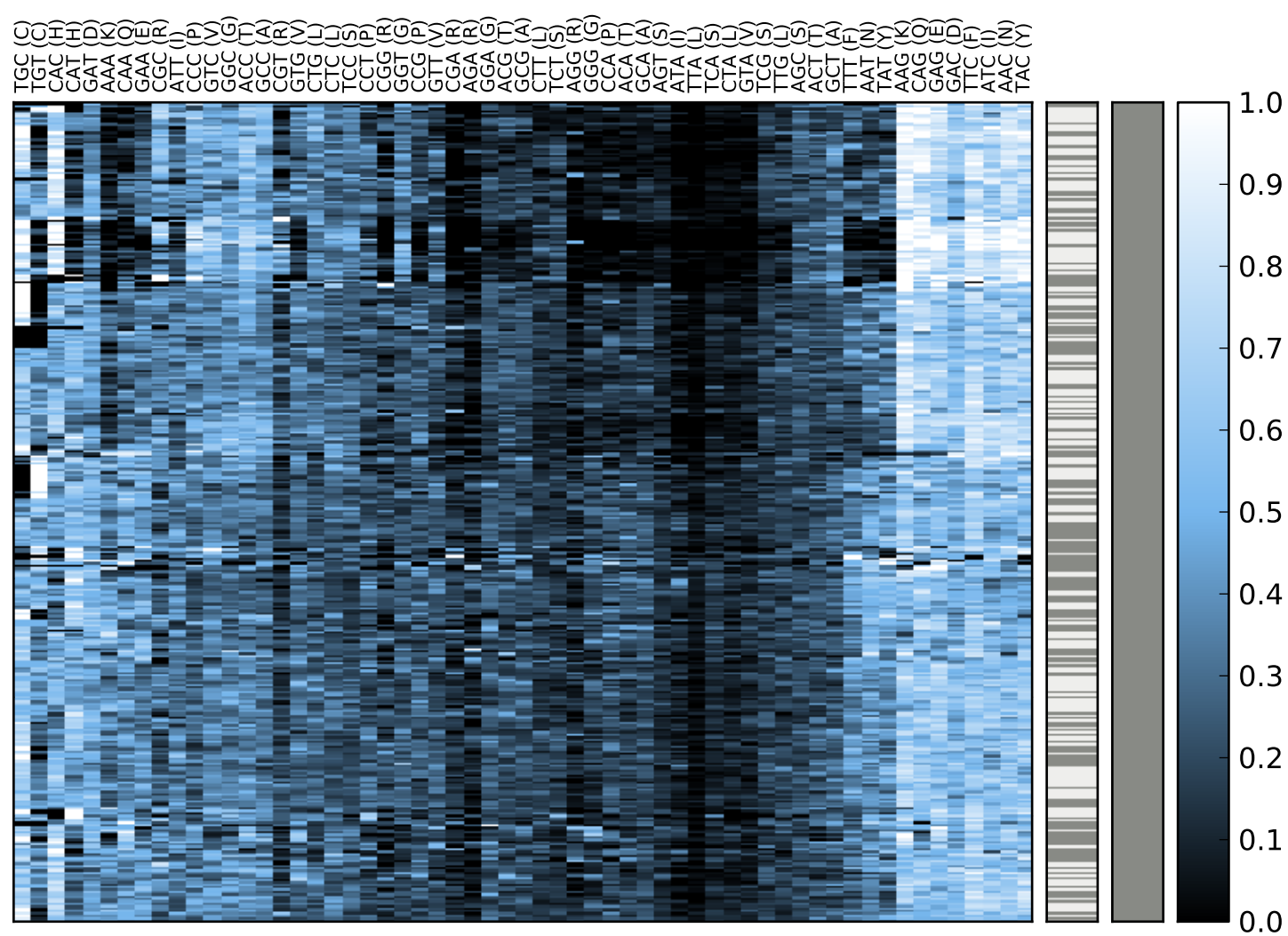

Supplement: Figure S7 — Shows a heat map of the codon usage feature matrix (), similar to Figure S1. (PDF) [file pone.0045869.s007.pdf]

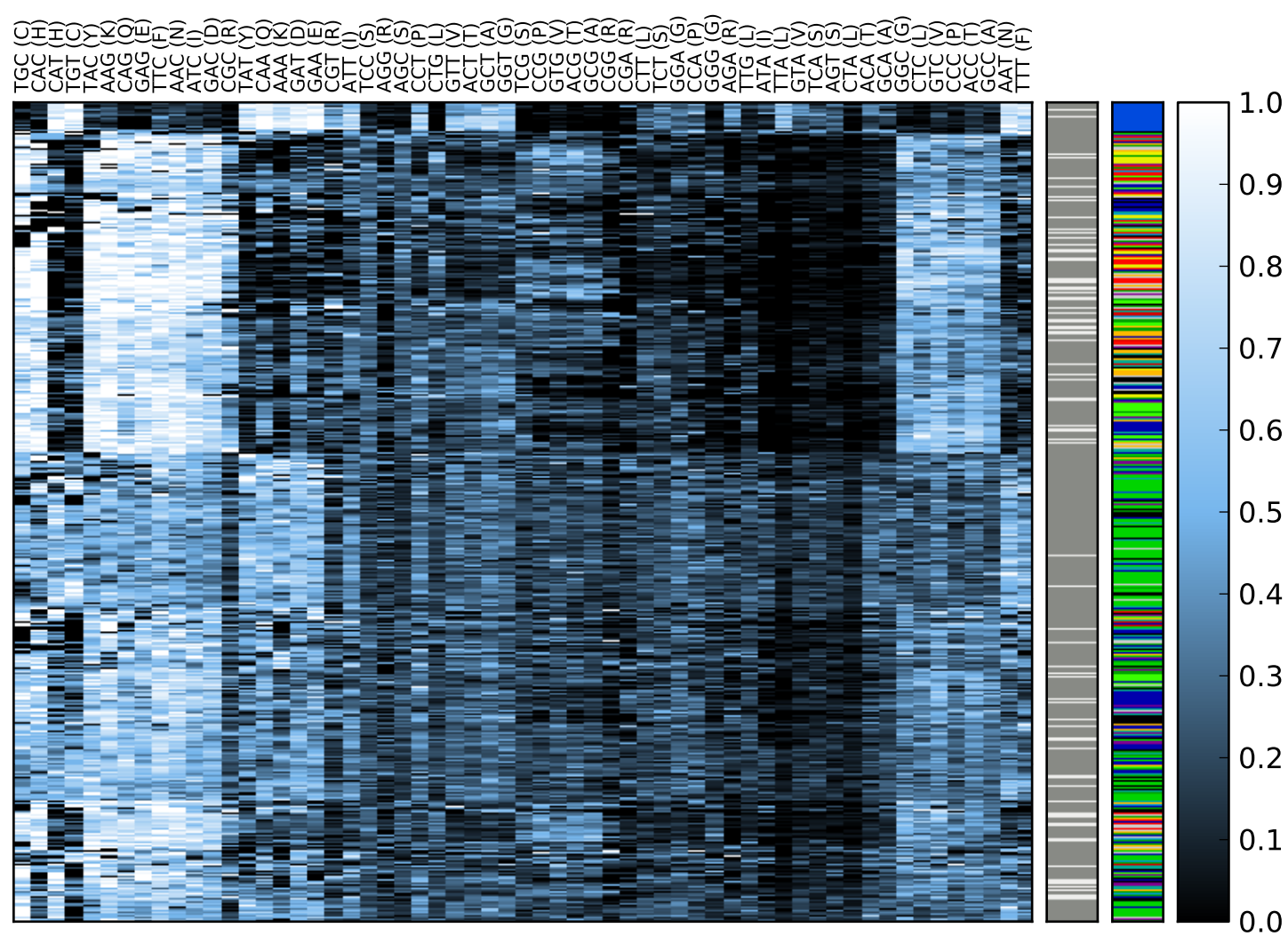

Supplement: Figure S8 — Shows a heat map of the codon usage feature matrix (), similar to Figure S1. (PDF) [file pone.0045869.s008.pdf]

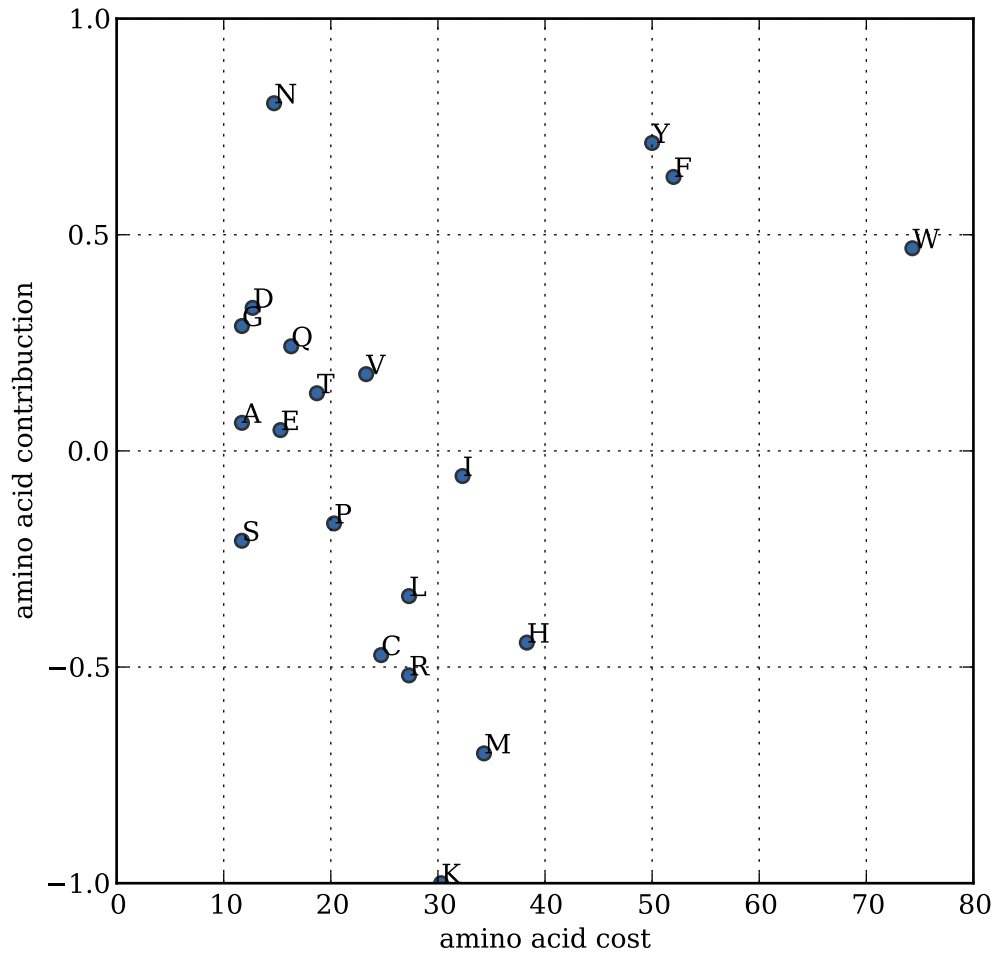

Supplement: Figure S9 — Shows the amino acid contributions of the classifier (-axis) versus amino acid costs (-axis). A correlation is observed for the non-aromatic amino acids, suggesting a preference for “cheap amino acids for high-level secretion. (PDF) [file pone.0045869.s009.pdf]

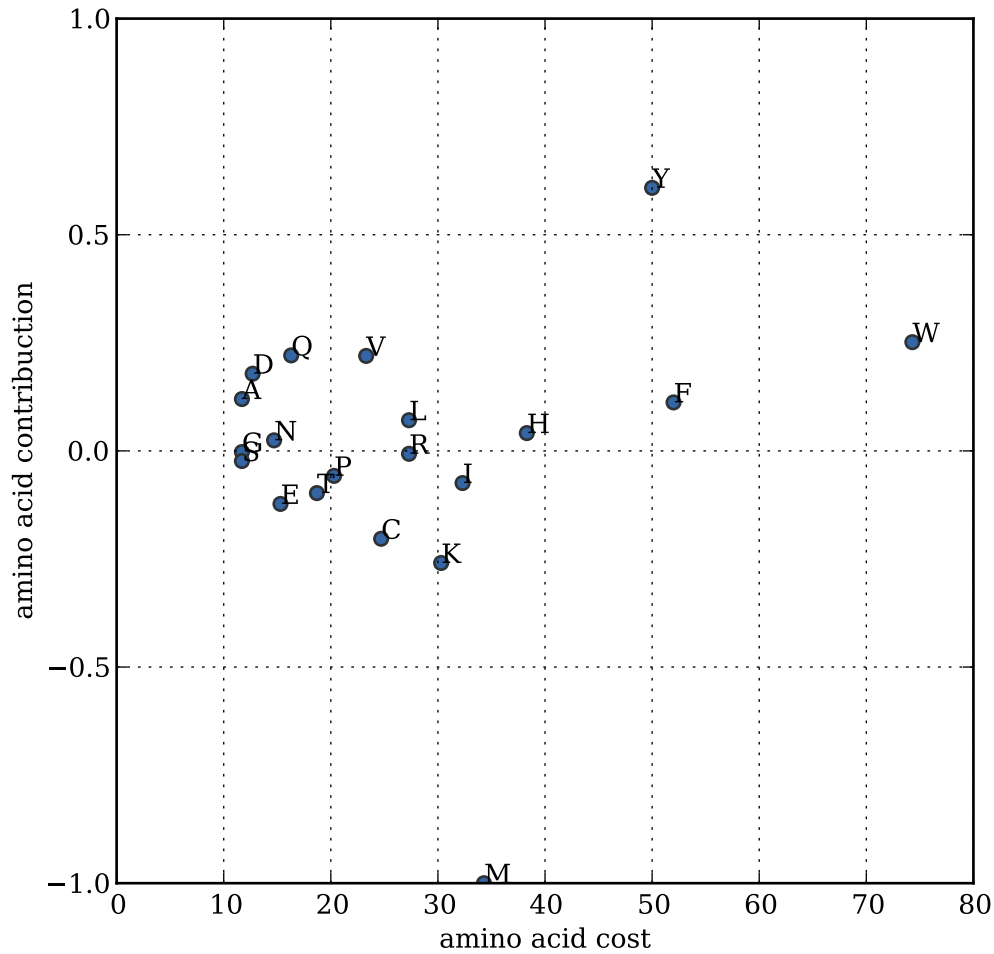

Supplement: Figure S10 — Shows the amino acid contributions of the classifier (-axis) versus amino acid costs (-axis). (PDF) [file pone.0045869.s010.pdf]

hom data set

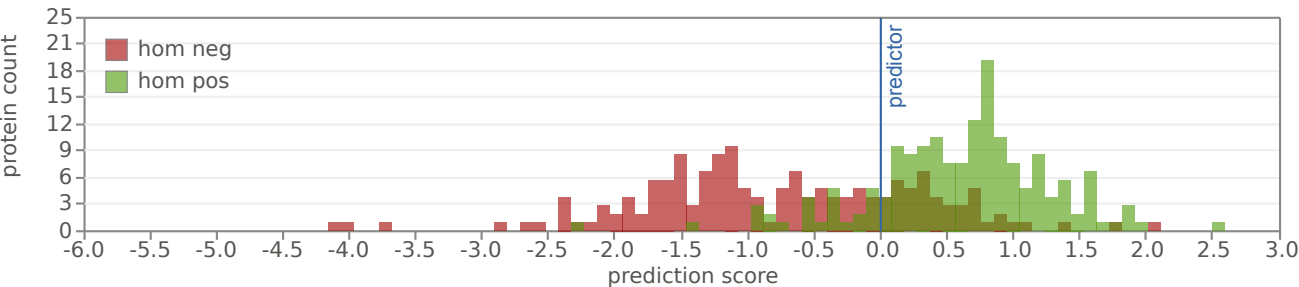

*Aspergillus niger* genome

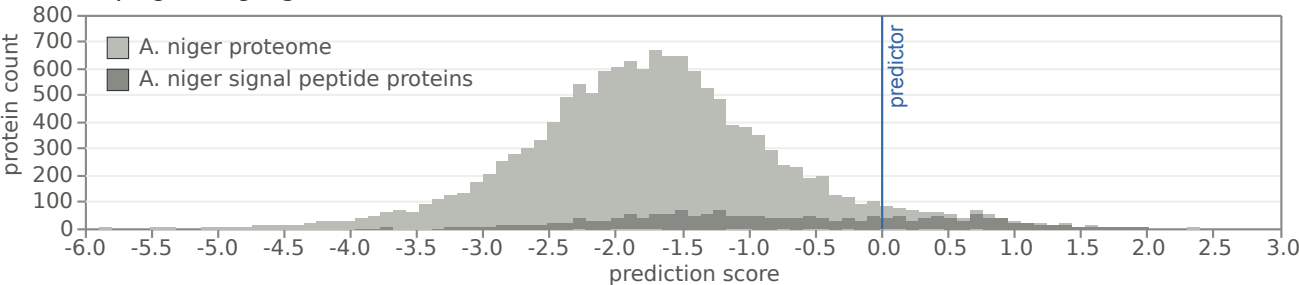

Supplement: Figure S11 — Shows the classifier outcomes of the protein composition classifier. A) The histogram shows the classifier outcomes for the data set with the negatively labeled proteins in red and the positively labeled proteins in green. Note that the classifier is trained using the same data set. B) The histogram shows the classifier outcomes for the A. niger proteome in light grey. The subset of the proteome that contains a predicted signal peptide (SignalP 3.0) is shown in dark grey. (PDF) [file pone.0045869.s011.pdf]
